# Evolved increases in hemoglobin-oxygen affinity and the Bohr effect coincided with the aquatic specialization of penguins

**DOI:** 10.1073/pnas.2023936118

**Published:** 2021-03-22

**Authors:** Anthony V. Signore, Michael S. Tift, Federico G. Hoffmann, Todd. L. Schmitt, Hideaki Moriyama, Jay F. Storz

**Affiliations:** ^a^School of Biological Sciences, University of Nebraska, Lincoln, NE 68588;; ^b^Department of Biology and Marine Biology, University of North Carolina, Wilmington, NC 28403;; ^c^Department of Biochemistry, Molecular Biology, Entomology, and Plant Pathology, Mississippi State University, Starkville, MS 39762;; ^d^Institute for Genomics, Biocomputing and Biotechnology, Mississippi State University, Starkville, MS 39762;; ^e^Veterinary Services, SeaWorld of California, San Diego, CA 92109

**Keywords:** hemoglobin, hypoxia, penguins, Bohr effect, adaptation

## Abstract

Dive capacities of air-breathing vertebrates are dictated by onboard O_2_ stores, suggesting that physiologic specialization of diving birds such as penguins may have involved adaptive changes in convective O_2_ transport. It has been hypothesized that increased hemoglobin (Hb)-O_2_ affinity improves pulmonary O_2_ extraction and enhances the capacity for breath-hold diving. To investigate evolved changes in Hb function associated with the aquatic specialization of penguins, we integrated comparative measurements of whole-blood and purified native Hb with protein engineering experiments based on site-directed mutagenesis. We reconstructed and resurrected ancestral Hb representing the common ancestor of penguins and the more ancient ancestor shared by penguins and their closest nondiving relatives (order Procellariiformes, which includes albatrosses, shearwaters, petrels, and storm petrels). These two ancestors bracket the phylogenetic interval in which penguin-specific changes in Hb function would have evolved. The experiments revealed that penguins evolved a derived increase in Hb-O_2_ affinity and a greatly augmented Bohr effect (i.e., reduced Hb-O_2_ affinity at low pH). Although an increased Hb-O_2_ affinity reduces the gradient for O_2_ diffusion from systemic capillaries to metabolizing cells, this can be compensated by a concomitant enhancement of the Bohr effect, thereby promoting O_2_ unloading in acidified tissues. We suggest that the evolved increase in Hb-O_2_ affinity in combination with the augmented Bohr effect maximizes both O_2_ extraction from the lungs and O_2_ unloading from the blood, allowing penguins to fully utilize their onboard O_2_ stores and maximize underwater foraging time.

## Significance

In diving birds like penguins, physiologic considerations suggest that increased hemoglobin (Hb)-O_2_ affinity may improve pulmonary O_2_ extraction and enhance dive capacity. We integrated experimental tests on whole-blood and native Hbs of penguins with protein engineering experiments on reconstructed ancestral Hbs. The experiments involving ancestral protein resurrection enabled us to test for evolved changes in Hb function in the stem lineage of penguins after divergence from their closest nondiving relatives. We demonstrate that penguins evolved an increased Hb-O_2_ affinity in conjunction with a greatly augmented Bohr effect (i.e., reduction in Hb-O_2_ affinity at low pH) that should maximize pulmonary O_2_ extraction without compromising O_2_ delivery at systemic capillaries.

In air-breathing vertebrates, diving capacities are dictated by onboard O_2_ stores and the efficiency of O_2_ use in metabolizing tissues (1). In fully aquatic taxa, selection to prolong breath-hold submergence and underwater foraging time may have promoted adaptive changes in multiple components of the O_2_ transport pathway, including oxygenation properties of hemoglobin (Hb). Vertebrate Hb is a tetrameric protein that is responsible for circulatory O_2_ transport, loading O_2_ in pulmonary capillaries and unloading O_2_ in the systemic circulation via quaternary structural shifts between a high-affinity (predominately oxygenated) relaxed (R) state and a low-affinity (predominately deoxygenated) tense (T) state (2). While this mechanism of respiratory gas transport is conserved in all vertebrate Hbs, amino acid variation in the constituent α- and β-type subunits may alter intrinsic O_2_ affinity and the responsiveness to changes in temperature, red cell pH, and red cell concentrations of allosteric cofactors (nonheme ligands that modulate Hb-O_2_ affinity by preferentially binding and stabilizing the deoxy T conformation) (3, 4).

While the quantity of Hb is typically increased in the blood of diving birds and mammals compared with their terrestrial relatives, there is no consensus on whether evolved changes in Hb-O_2_ affinity have contributed to enhanced diving capacity (1). It has been hypothesized that increased Hb-O_2_ affinity may improve pulmonary O_2_ extraction in diving mammals, thereby enhancing diving capacity (5), but more comparative data are needed to assess evidence for an adaptive trend (6, 7). Experimental measurements on whole blood suggest that the emperor penguin (*Aptenodytes forsteri*) may have a higher blood-O_2_ affinity relative to nondiving waterbirds, a finding that has fostered the view that this is a property characterizing penguins as a group (8–10). However, blood-O_2_ affinity is a highly plastic trait that is influenced by changes in red cell metabolism and acid-base balance, so measurements on purified Hb under standard assay conditions are needed to assess whether observed species differences in blood-O_2_ affinity stem from genetically based changes in the oxygenation properties of Hb. Moreover, even if species differences in Hb-O_2_ affinity are genetically based, comparative data from extant taxa do not reveal whether observed differences are attributable to a derived increase in penguins, a derived reduction in their nondiving relatives, or a combination of changes in both directions.

**Fig. 1.**
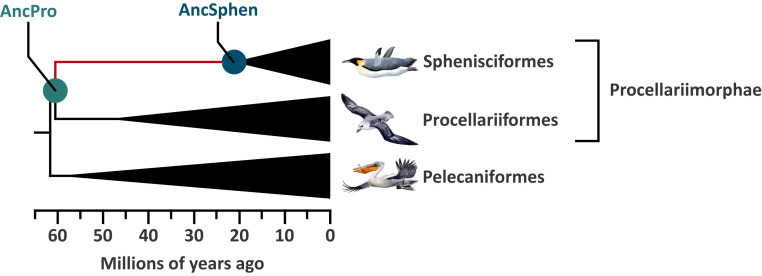
Diagrammatic phylogeny showing the relationship among Sphenisciformes (penguins), Procellariiformes, and Pelecaniformes. Ancestral Hbs were reconstructed for the two indicated nodes: AncSphen and AncPro (the super order that contains Sphenisciformes and Procellariiformes). Divergence times are adapted from Claramunt and Cracraft (56).

To investigate evolved changes in Hb function associated with the aquatic specialization of penguins, we integrated experimental measurements of whole-blood and purified native Hb with evolutionary analyses of globin sequence variation. To characterize the mechanistic basis of evolved changes in Hb function in the stem lineage of penguins, we performed protein engineering experiments on reconstructed and resurrected ancestral Hb representing the common ancestor of penguins and the more ancient ancestor shared by penguins and their closest nondiving relatives (order Procellariiformes, which includes albatrosses, shearwaters, petrels, and storm petrels) (Fig. 1). These two ancestors bracket the phylogenetic interval in which penguin-specific changes in Hb function would have evolved.

## Results and Discussion

### O_2_-Binding Properties of Penguin Whole-Blood and Purified Hb.

**Fig. 2.**
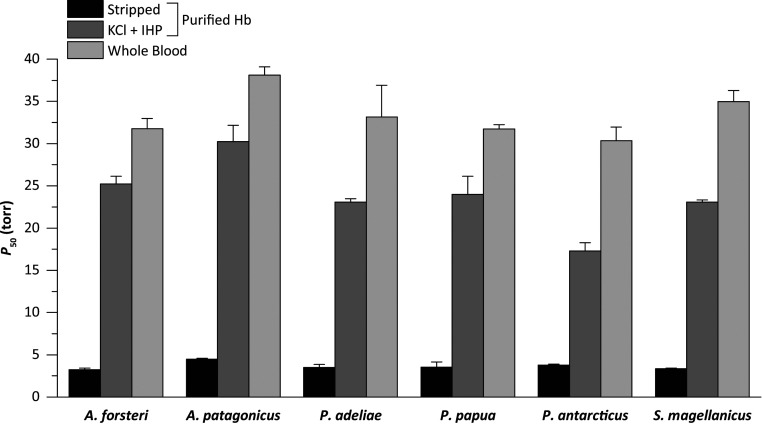
P_50_ values for penguin whole-blood and purified Hb at 37 °C, in the absence (stripped) and presence of 100 mM KCl and 0.2 mM IHP (+KCl +IHP). The higher the P_50_, the lower the Hb-O_2_ affinity. Whole-blood P_50_ values are presented as mean ± SE (*n* = 3). Purified Hb P_50_ values are derived from plots of logP_50_ vs. pH in which a linear regression was fit to estimate P_50_ at exactly pH 7.40 (± SE of the regression estimate).

Using blood samples from multiple individuals of six penguin species, we measured the partial pressure of O_2_ (PO_2_) at 50% saturation (P_50_) for whole-blood and purified Hb in the absence (stripped) and presence of allosteric cofactors (+KCl +IHP [inositol hexaphosphate]) (Fig. 2). Whole-blood P_50_ values were similar across all penguins, averaging 33.3 ± 1.1 torr (Fig. 2 and *SI Appendix*, Table S1), consistent with previously published data for emperor, Adélie, chinstrap, and gentoo penguins (8, 9, 11). Similarly, measured O_2_ affinities for purified Hb exhibited very little variation among species in both the presence and absence of allosteric cofactors (Fig. 2 and *SI Appendix*, Table S1). Penguins express a single Hb isoform during postnatal life (HbA), in contrast to the majority of other bird species that express one major and one minor isoform (HbA and HbD, respectively) (12, 13). The lack of variation in Hb-O_2_ affinity among penguins is consistent with the low level of amino acid variation in the α- and β-chains (*SI Appendix*, Fig. S1). The experiments revealed that penguin Hb exhibits a remarkably large shift in the magnitude of the Bohr effect (i.e., reduced Hb-O_2_ affinity in response to reduced pH) with the addition of allosteric cofactors (*SI Appendix*, Table S1). The average Bohr effect of penguin Hbs more than doubles with the addition of allosteric cofactors, from −0.21 ± 0.03 to −0.53 ± 0.04 (*SI Appendix*, Table S1).

Our experimental results indicate that penguins have a generally higher Hb-O_2_ affinity than other birds (12, 14–22), consistent with previous suggestions based on measurements of whole blood (8, 9, 23–25). Whole-blood O_2_ affinities of the six examined penguin species (30.4 to 38.1 torr at 37 °C, pH 7.40) were uniformly higher than that from a representative member of Procellariiformes, the southern giant petrel (*Macronectes giganteus*; 42.5 torr at 38 °C, pH 7.40) (9). Similarly, numerous high-altitude bird species have convergently evolved increased Hb-O_2_ affinities (17, 18, 21), which appears to be adaptive because it helps safeguard arterial O_2_ saturation despite the reduced PO_2_ of inspired air (26–28). The difference in blood P_50_ values between penguins and the southern giant petrel is generally much greater in magnitude than differences in Hb P_50_ between closely related species of low- and high-altitude birds (14–18, 20–22). Similar to the case of other diving vertebrates (29), the Bohr effect of penguin Hb also greatly exceeds typical avian values.

### Ancestral Protein Resurrection.

**Fig. 3.**
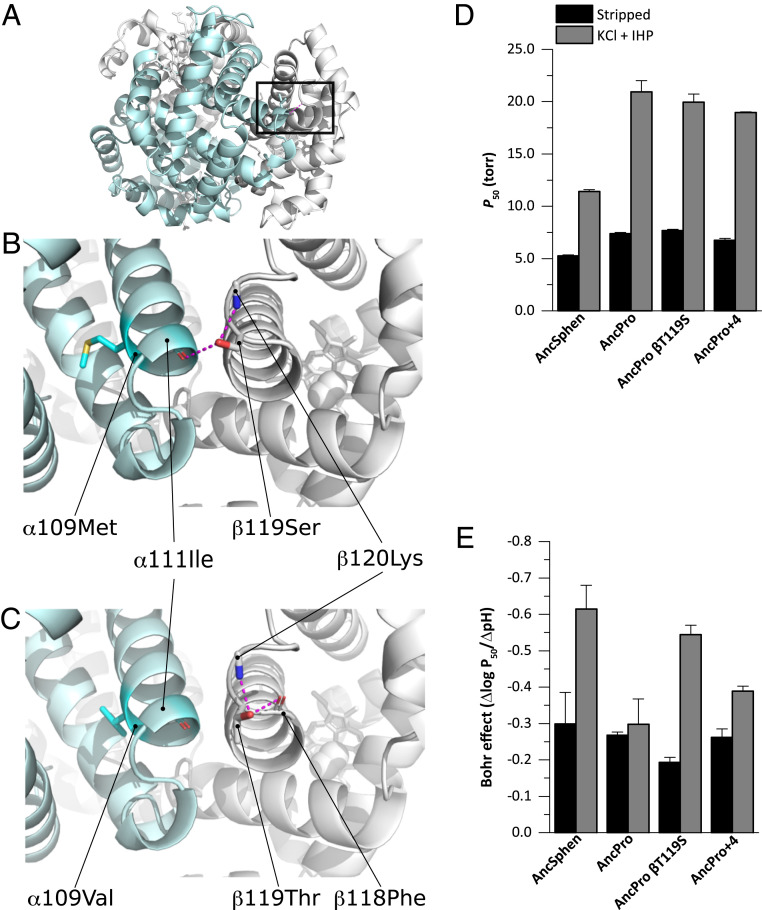
Structural (*A*–*C*) and physiological (*D* and *E*) effects of amino acid substitutions in the reconstructed Hb proteins of the penguin ancestor (AncSphen) and the last common ancestor penguins shared with Procellariiformes (AncPro). (*A*) Molecular model of the AncSphen Hb tetramer, with the black box indicating the regions highlighted in *B* and *C*. (*B*) Molecular model of AncSphen Hb showing intersubunit stabilizing H bonds (pink) between β119Ser and both α111Ile and β120Lys. (*C*) Molecular model of AncPro Hb showing that replacement of β119Ser with Thr removes the intersubunit stabilizing H bonds. (*D*) Hb-O_2_ affinity (as measured by P_50_) of AncSphen, AncPro, and two mutant rHbs with penguin-specific amino acid replacements introduced on the AncPro background: AncProβ119Ser and AncPro+4. See the text for an explanation of the choice of candidate sites for mutagenesis experiments. Measurements were performed on Hb solutions (0.1 mM Hb in 0.1 M Hepes/0.5 mM EDTA) at 37 °C in the absence (stripped) and presence of +KCl +IHP. P_50_ values are derived from plots of logP_50_ vs. pH, in which a linear regression was fit to estimate P_50_ at exactly pH 7.40 (± SE of the regression estimate). (*E*) Bohr coefficients (Δlog P_50_/ΔpH) were estimated from plots of logP_50_ vs. pH in which the Bohr effect is represented by the slope of the linear regression (± SE of the slope estimate).

In principle, the observed difference in Hb-O_2_ affinity between penguins and their closest nondiving relatives could be explained by a derived increase in Hb-O_2_ affinity in the penguin lineage (the generally assumed adaptive scenario), a derived reduction in the stem lineage of Procellariiformes (the nondiving sister group), or a combination of changes in both directions. To test these alternative hypotheses, we reconstructed the Hbs of the common ancestor of penguins (AncSphen) and the more ancient common ancestor of Procellariimorphae (the superorder comprising Sphenisciformes [penguins] and Procellariiformes; AncPro) (Fig. 1 and *SI Appendix*, Figs. S2–S4). We then recombinantly expressed and purified the ancestral Hb to perform in vitro functional tests. Measurements of O_2_ equilibrium curves revealed that the AncSphen Hb has a significantly higher O_2_ affinity than AncPro Hb (Fig. 3), indicating that penguins evolved a derived increase in Hb-O_2_ affinity. In the presence of allosteric cofactors, the P_50_ of AncSphen is much lower (i.e., O_2_ affinity is higher) compared to AncPro (11.8 vs. 20.2 torr). Much like the evolved increases in Hb-O_2_ affinity in high-altitude birds (18, 20–22, 30), the increased O_2_ affinity of penguin Hb is attributable to an increase in intrinsic affinity rather than to reduced responsiveness to allosteric cofactors, as the Hb-O_2_ affinity difference between AncSphen and AncPro persists in the presence and absence of Cl^−^ and IHP (Fig. 3).

In addition to the derived increase in Hb-O_2_ affinity, comparisons between AncSphen and AncPro also revealed that the Hb of penguins evolved an enhanced responsiveness to pH (Bohr effect). Under stripped conditions, the Bohr effect of AncSphen and AncPro (−0.30 ± 0.09 and −0.27 ± 0.1, respectively) were highly similar to each other and similar to values measured in native penguin Hb under the same conditions (Fig. 3*E* and *SI Appendix*, Table S1). However, in the presence of allosteric cofactors, the Bohr effect of AncSphen increased more than two-fold (similar to that of native penguin Hb), whereas that of AncPro showed little change (Fig. 3*E*), demonstrating that penguins evolved an increased cofactor-linked Bohr effect following divergence from their nondiving relatives. An increased Hb-O_2_ affinity is expected to reduce the gradient for O_2_ diffusion from systemic capillaries to the cells of metabolizing tissues, and an increased Bohr effect can compensate for this by reducing Hb-O_2_ affinity at low pH, thereby promoting O_2_ unloading in acidified tissues. A similar augmentation of the Bohr effect was recently documented in the Hb of high-altitude Tibetan canids (31). In summary, the Hb of penguins evolved an increase in O_2_ affinity and an enhanced Bohr effect in association with other physiologic and morphologic specializations for a more fully aquatic existence.

### Tests of Positive Selection.

Given that joint increases in the O_2_ affinity and Bohr effect of penguin Hb represent derived character states, we performed a molecular evolution analysis to test for evidence of positive selection in the α- and β-globin genes. Specifically, we tested for an accelerated rate of amino acid substitution in the stem lineage of penguins (the branch connecting AncPro to AncSphen) using the branch sites test. This test revealed no evidence for an accelerated rate of amino acid substitution in the stem lineage of penguins (*SI Appendix*, Table S2), and a clade test revealed no significant variation in the substitution rate among different penguin lineages (*SI Appendix*, Table S3). Thus, if the increased Hb-O_2_ affinity of penguins represents an adaptation that evolved via positive selection, the nature of the causative changes did not produce a detectable statistical signature in the α- and β-type globin genes.

### Molecular Modeling.

We used molecular modeling to identify which specific amino acid substitutions may be responsible for the increased Hb-O_2_ affinity of AncSphen relative to AncPro. Of the 17 amino acid substitutions that distinguish AncSphen and AncPro, our analyses identified four substitutions that could potentially alter O_2_-binding properties. The substitution Thrβ119Ser in the branch leading to AncSphen affects the stabilization of R state (oxygenated) Hb. Specifically, the hydroxyl group of β119Ser in helix G is oriented toward the subunit interface by forming a hydrogen-bond with β120Lys, which permits an intersubunit contact with α111Ile (Fig. 3 *A* and *B*). This bond between β119Ser and α111Ile stabilizes the R state conformation by clamping the intersubunit motions, which is predicted to increase Hb-O_2_ affinity by raising the free energy of the oxygenation-linked allosteric R→T transition in quaternary structure. Additionally, our model identified three other amino acid substitutions—αA138S, βA51S, and βI55L—that create intersubunit contacts and further stabilize the R state conformation.

### Testing Causative Substitutions.

To test model-based predictions about the specific substitutions that are responsible for the increased O_2_ affinity of penguin Hb, we used site-directed mutagenesis to introduce combinations of mutations at four candidate sites on the AncPro background. We first tested the effect of a single mutation, whereby β119Thr was replaced with Ser (AncProβT119S). We then tested the net effect of mutations at all four sites on the AncPro background (AncPro+4: αA138S, βA51S, βI55L, and βT119S). The protein engineering experiments revealed that βT119S produced a negligible individual effect on Hb-O_2_ affinity when introduced on the AncPro background, but produced an appreciable increase in the Bohr effect (Fig. 3 *D* and *E*). The four mutations in combination produced a modest increase in Hb-O_2_ affinity and a more pronounced increase in the Bohr effect, but they did not fully recapitulate the observed differences between AncPro and AncSphen in either of these properties (Fig. 3). These data suggest the evolved functional changes in penguin Hb must be attributable to the net effect of multiple amino acid substitutions at structurally disparate sites.

### Adaptive Significance of Increased Hb-O_2_ Affinity.

The key to extending dive times for aquatic vertebrates is to increase O_2_ carrying capacity while keeping metabolic O_2_ demands as low as possible during breath-hold submergence. Submergence induces intense bradycardia and peripheral vasoconstriction, which conserves finite O_2_ stores for tissues that are intolerant to hypoxia (i.e., the central nervous system and heart) (32–35). O_2_ stores are typically increased in diving vertebrates via increased blood volume, increased blood Hb concentration, increased myoglobin concentration in skeletal muscle, increased muscle mass, and, occasionally, increased diving lung volume (1). As deep-diving cetaceans and pinnipeds exhale before submergence, their lungs account for less than 10% of total O_2_ stores (1, 36). This reduction in diving lung volume reduces gaseous N_2_ and O_2_, which presumably limits decompression sickness. Conversely, as penguins inhale at the onset of a dive, their diving lung volume accounts for a much larger percentage of total O_2_ stores (19% for emperor penguins and 45% for Adélie penguins) (1, 37). Indeed, in diving emperor penguins, O_2_ extraction from pulmonary stores is continuous during submergence (38, 39). An elevated Hb-O_2_ affinity (such as that found in penguins) can maximize O_2_ extraction from pulmonary stores, as greater blood O_2_ saturation can be achieved at any given parabronchial PO_2_ value. However, while increased Hb-O_2_ affinity may confer more complete transfer of O_2_ from the lungs to the blood, it can inhibit subsequent O_2_ transfer from the blood to the tissues. Despite this, emperor penguins deplete their circulatory stores almost completely during extended dives, as their end-of-dive venous PO_2_ can be as low as 1 to 6 torr (38). The enhanced Bohr effect of penguin Hb should improve O_2_ transport to working (acidic) tissues, allowing more complete O_2_ unloading of the blood. We suggest that this modification works in tandem with increased Hb-O_2_ affinity to maximize both O_2_ extraction from the lungs and O_2_ unloading from the blood, allowing penguins to fully utilize their onboard O_2_ stores and maximize underwater foraging time.

## Materials and Methods

### Blood Collection.

We collected blood from 18 individual penguins representing six species: *A. forsteri*, *Aptenodytes patagonicus*, *Pygoscelis adeliae*, *Pygoscelis papua*, *Pygoscelis antarcticus*, and *Spheniscus magellanicus* (*n* = 3 individuals per species). All birds were sampled during routine health checks at SeaWorld of California. Blood was collected by venipuncture of the jugular vein using BD Vacutainer Safety-Lok blood collection set with a 21 G × 3/4” (0.8 × 19 mm) needle attached to a heparin blood collection tube (BD). A subsample of whole blood (200 µL) was set aside for oxygen equilibrium curves (see below), and the remaining blood was centrifuged at 5,000 × *g* for 15 min. Plasma, buffy coat, and hematocrit fractions from the centrifuged samples were immediately placed in separate tubes and flash-frozen at −80 °C for future analyses.

### Sequencing of Penguin Globin Genes.

Globin gene sequencing was performed as described previously (40). In brief, RNA was extracted from ∼100 µL of flash frozen erythrocytes using the Qiagen RNeasy Universal Plus Mini Kit. cDNA was synthesized from freshly prepared RNA using SuperScript IV reverse transcriptase (Invitrogen). Gene-specific primers used to amplify the α- and β-type globin transcripts were designed from the 5′ and 3′ flanking regions of all publicly available penguin globin genes. PCR reactions were conducted using 1 mL of cDNA template in 0.2-mL tubes containing 25 µL of reaction mixture (0.5 µL of each dNTP [2.5 mM], 2.5 µL of 10X Reaction Buffer [Invitrogen], 0.75 µL of 50 mM MgCl_2_, 1.25 µL of each primer [10 pmol/µL], 1 µL of Taq polymerase [Invitrogen], and 16.75 µL of ddH_2_O), using an Eppendorf Mastercycler gradient thermocycler. Following a 5-min denaturation period at 94 °C, the desired products were amplified using a cycling profile of 94 °C for 30 s, 53 to 65 °C for 30 s, and 72 °C for 45 s for 30 cycles, followed by a final extension period of 5 min at 72 °C. Amplified products were run on a 1.5% agarose gel, and bands of the correct size were subsequently excised and purified using Zymoclean Gel DNA recovery columns (Zymo Research). Gel-purified PCR products were ligated into pCR4-TOPO vectors using the TOPO TA Cloning Kit and then transformed into One Shot TOP10 chemically competent *Escherichia coli* (Thermo Fisher Scientific). Three to six transformed colonies were cultured in 5 mL of LB medium, and plasmids were subsequently purified with the GeneJET Plasmid Midiprep Kit (Thermo Fisher Scientific). Purified plasmids were sequenced by Eurofins Genomics.

### Sequence Analyses.

Genomic sequences containing the complete α- and β-globin gene clusters for the emperor penguin (*A. forsteri*), Adélie penguin (*P. adeliae*), northern fulmar (*Fulmarus glacialis*), band-rumped storm petrel (*Hydrobates castro*), southern giant petrel (*M. giganteus*), flightless cormorant (*Nannopterum harrisi*), crested ibis (*Nipponia nippon*), and little egret (*Egretta garzetta*) were obtained from GenBank. The α- and β-globin gene clusters from the remaining 19 extant penguin species were obtained from GigaDB (41). Coding sequences of α- and β-globin genes extracted from these genomic sequences were combined with the newly generated cDNA sequences mentioned above (*SI Appendix*, Fig. S2). Sequences were aligned using MUSCLE (42) and then used to estimate phylogenetic trees as described previously (40). In brief, the best-fitting codon substitution model and initial tree search were estimated using IQ-TREE with the options -st CODON, -m TESTNEW, -allnni, and -bnni (43, 44). Initial trees were then subjected to 1,000 μLtrafast bootstrap replicates (45). Bootstrap consensus trees (*SI Appendix*, Fig. S3) were used to estimate ancestral globin sequences using IQ-TREE with the option -asr (*SI Appendix*, Figs. S2 and S4).

### Selection Analyses.

We tested for selection in the evolution of the penguins’ α- and β-globin genes in a maximum likelihood framework with the codon-based models implemented in the codeml program from the PAML v4.9 suite (46), using the phylogenetic trees described above. We used the branch site and clade models to examine variations in ω, the ratio of the rate of nonsynonymous substitutions per nonsynonymous site, dN, to the rate of synonymous substitutions per synonymous site, dS. We used branch site model A (47, 48) to test for positive selection in the branch connecting AncPro to AncSphen (the stem lineage of penguins) (*SI Appendix*, Table S2), and used the clade C model (49) to test for selection in the penguin clade using M2a_rel from Weadick and Chang (50) as the null model (*SI Appendix*, Table S3).

### Molecular Modeling.

Structural modeling was performed on the SWISS MODEL server (51) using graylag goose Hb in oxy form (PDB ID code 1FAW). AncPro Hb and AncSphen Hb had QMEAN values of –0.61 and –0.65, respectively. A root mean square distance (RMSD) of the main chain between template and model values <0.09 Å was considered usable (52). Structural mining and preparation of graphics were performed using the PyMOL Molecular Graphics System, version 2.3.2 (Schrödinger). Hydrogen bond listing was performed using a PyMOL script list_hb.py (Robert L. Campbell, Biomedical and Molecular Sciences, Queen's University). The interface binding energy was calculated by the ePISA server (53).

### Construction of Hb Expression Vectors.

Reconstructed ancestral globins were synthesized by GeneArt Gene Synthesis (Thermo Fisher Scientific) after optimizing the nucleotide sequences in accordance with *E. coli* codon preferences. The synthesized globin gene cassette was cloned into a custom pGM vector system along with the methionine aminopeptidase (MAP) gene, as described previously (54). We engineered the Thrβ119Ser substitution by whole-plasmid amplification using mutagenic primers and Phusion High-Fidelity DNA Polymerase (New England BioLabs), phosphorylation with T4 Polynucleotide Kinase (New England BioLabs), and circularization with the NEB Quick Ligation Kit (New England BioLabs). All site-directed mutagenesis steps were performed using the manufacturer’s recommended protocol. Each plasmid was verified with DNA sequencing by Eurofins Genomics.

### Expression and Purification of Recombinant Hb.

Recombinant Hb expression was carried out in the *E. coli* JM109 (DE3) strain as described previously (15, 54, 55). Bacterial cell lysates were loaded onto a HiTrap SP HP anion exchange column (GE Healthcare) and then equilibrated with 50 mM Hepes/0.5 mM EDTA (pH 7.0) and eluted with a linear gradient of 0 to 0.25 M NaCl. Hb-containing fractions were then loaded onto a HiTrap Q HP cation exchange column (GE Healthcare) equilibrated with 20 mM Tris⋅HCl/0.5 mM EDTA (pH 8.6) and eluted with a linear pH gradient of 0 to 0.25 M NaCl. Eluted Hb factions were concentrated using Amicon Ultra-4 Centrifugal Filter Units (EMD Millipore).

### Sample Preparation for O_2_ Equilibrium Curves.

Fresh whole blood was diluted 1:15 with each individual’s own plasma, and O_2_ equilibrium curves were measured immediately after sampling. To obtain stripped hemolysate, 100 μL of centrifuged red blood cells were added to a 5× volume of 0.01 M Hepes/0.5 mM EDTA buffer (pH 7.4), followed by a 30-min incubation on ice to lyse the red blood cells. NaCl was added to a final concentration of 0.2 M, and samples were centrifuged at 20,000 × *g* for 10 min to remove cell debris. Hemolysate supernatants and purified recombinant Hb were similarly desalted by passing through a PD-10 desalting column (GE Healthcare) equilibrated with 25 mL of 0.01 M Hepes/0.5 mM EDTA (pH 7.4). Eluates were concentrated using Amicon Ultra-4 Centrifugal Filter Units (EMD Millipore). From these concentrated samples, Hb solutions (0.1 mM Hb in 0.1 M Hepes/0.05 M EDTA buffer) were prepared in the absence (stripped) and the presence of 0.1 M KCl and 0.2 mM inositol hexaphosphate (+KCl +IHP). Stripped and +KCl +IHP treatments were prepared at three different pH values (for a total of six treatments per Hb sample). Working solutions were adjusted with NaOH to as close to pH 7.2, 7.4, or 7.6 as possible, and then pH was precisely measured with an Orion Star A211 pH Meter and Orion PerpHecT ROSS Combination pH Micro Electrode (Thermo Fisher Scientific).

### Measuring O_2_-Binding Properties.

O_2_ equilibrium curves were measured using a blood oxygen-binding system (BOBS; Loligo Systems) at 37 °C. The pH of whole-blood samples was set by measuring curves in the presence of 45 torr CO_2_, whereas the pH of Hb solutions was set with Hepes buffer (see above). Each whole-blood sample and Hb solution was sequentially equilibrated with an array of oxygen tensions (PO_2_), while the sample absorbance was continually monitored at 430 nm (deoxy peak) and 421 nm (oxy/deoxy isobestic point). Each equilibration step was considered complete when the absorbance at 430 nm had stabilized (2 to 4 min). Only PO_2_ values yielding 30 to 70% Hb O_2_ saturation were used in subsequent analyses. Hill plots (log[fractional saturation/[1 − fractional saturation]] vs. logPO_2_) were constructed from these measurements. A linear regression was fitted to these plots and used to determine the PO_2_ at half-saturation (P_50_) and the cooperativity coefficient (n_50_), where the *x*-intercept and slope of the regression line represent the P_50_ and n_50_, respectively. Values for whole-blood samples (*n* = 3) are presented as mean ± SE. For Hb solutions, a linear regression was fit to plots of logP_50_ vs. pH, and the resulting equation was used to estimate P_50_ values at pH 7.40 (± SE of the regression estimate). We did not make direct comparisons between native Hb and recombinantly expressed Hb, because recombinant Hb often exhibits slightly lower P_50_ values due to an increased rate of autoxidation. Thus, all inferences are based on comparisons among native Hb samples from extant species or on comparisons between recombinant Hb samples representing reconstructed ancestors

## Supplementary Material

Supplementary File

## Data Availability

All study data are included in the main text and *SI Appendix*.

## References

[1] G. L. Kooyman, P. J. Ponganis, The physiological basis of diving to depth: Birds and mammals. Annu. Rev. Physiol. 60, 19–32 (1998). 9558452 10.1146/annurev.physiol.60.1.19

[2] M. F. Perutz, Stereochemistry of cooperative effects in haemoglobin. Nature 228, 726–739 (1970). 5528785 10.1038/228726a0

[3] M. F. Perutz, Species adaptation in a protein molecule. Mol. Biol. Evol. 1, 1–28 (1983). 6400645 10.1093/oxfordjournals.molbev.a040299

[4] J. F. Storz, Hemoglobin: Insights into Protein Structure, Function, and Evolution (Oxford University Press, 2019).

[5] G. K. Snyder, Respiratory adaptations in diving mammals. Respir. Physiol. 54, 269–294 (1983). 6369460 10.1016/0034-5687(83)90072-5

[6] A. V. Signore., Emergence of a chimeric globin pseudogene and increased hemoglobin oxygen affinity underlie the evolution of aquatic specializations in Sirenia. Mol. Biol. Evol. 36, 1134–1147 (2019). 30828717 10.1093/molbev/msz044 PMC6526914

[7] M. S. Tift, P. J. Ponganis, Time domains of hypoxia adaptation—elephant seals stand out among divers. Front. Physiol. 10, 677 (2019). 31214049 10.3389/fphys.2019.00677 PMC6558045

[8] J. U. Meir, P. J. Ponganis, High-affinity hemoglobin and blood oxygen saturation in diving emperor penguins. J. Exp. Biol. 212, 3330–3338 (2009). 19801437 10.1242/jeb.033761

[9] W. K. Milsom, K. Johansen, R. W. Millard, Blood respiratory properties in some Antarctic birds. Condor 75, 472–474 (1973).

[10] B. Wienecke, G. Robertson, R. Kirkwood, K. Lawton, Extreme dives by free-ranging emperor penguins. Polar Biol. 30, 133–142 (2007).

[11] C. Lenfant, G. L. Kooyman, R. Elsner, C. M. Drabek, Respiratory function of blood of the Adélie penguin Pygoscelis adeliae. Am. J. Physiol. 216, 1598–1600 (1969). 5786751 10.1152/ajplegacy.1969.216.6.1598

[12] M. T. Grispo., Gene duplication and the evolution of hemoglobin isoform differentiation in birds. J. Biol. Chem. 287, 37647–37658 (2012). 22962007 10.1074/jbc.M112.375600 PMC3488042

[13] J. C. Opazo., Gene turnover in the avian globin gene families and evolutionary changes in hemoglobin isoform expression. Mol. Biol. Evol. 32, 871–887 (2015). 25502940 10.1093/molbev/msu341 PMC4379397

[14] J. Projecto-Garcia., Repeated elevational transitions in hemoglobin function during the evolution of Andean hummingbirds. Proc. Natl. Acad. Sci. U.S.A. 110, 20669–20674 (2013). 24297909 10.1073/pnas.1315456110 PMC3870697

[15] Z. A. Cheviron., Integrating evolutionary and functional tests of adaptive hypotheses: A case study of altitudinal differentiation in hemoglobin function in an Andean sparrow, Zonotrichia capensis. Mol. Biol. Evol. 31, 2948–2962 (2014). 25135942 10.1093/molbev/msu234 PMC4209134

[16] S. C. Galen., Contribution of a mutational hot spot to hemoglobin adaptation in high-altitude Andean house wrens. Proc. Natl. Acad. Sci. U.S.A. 112, 13958–13963 (2015). 26460028 10.1073/pnas.1507300112 PMC4653164

[17] C. Natarajan., Convergent evolution of hemoglobin function in high-altitude Andean waterfowl involves limited parallelism at the molecular sequence level. PLoS Genet. 11, e1005681 (2015). 26637114 10.1371/journal.pgen.1005681 PMC4670201

[18] C. Natarajan., Predictable convergence in hemoglobin function has unpredictable molecular underpinnings. Science 354, 336–339 (2016). 27846568 10.1126/science.aaf9070 PMC5464326

[19] A. Kumar., Stability-mediated epistasis restricts accessible mutational pathways in the functional evolution of avian hemoglobin. Mol. Biol. Evol. 34, 1240–1251 (2017). 28201714 10.1093/molbev/msx085 PMC5400398

[20] A. Jendroszek., Allosteric mechanisms underlying the adaptive increase in hemoglobin-oxygen affinity of the bar-headed goose. J. Exp. Biol. 221, jeb185470 (2018). 30026237 10.1242/jeb.185470 PMC6176913

[21] X. Zhu., Divergent and parallel routes of biochemical adaptation in high-altitude passerine birds from the Qinghai-Tibet Plateau. Proc. Natl. Acad. Sci. U.S.A. 115, 1865–1870 (2018). 29432191 10.1073/pnas.1720487115 PMC5828625

[22] C. Natarajan., Molecular basis of hemoglobin adaptation in the high-flying bar-headed goose. PLoS Genet. 14, e1007331 (2018). 29608560 10.1371/journal.pgen.1007331 PMC5903655

[23] E. H. Christensen, B. Dill, Oxygen dissociation curves of bird blood. J. Biol. Chem. 109, 443–448 (1935).

[24] F. H. Baumann, R. Baumann, A comparative study of the respiratory properties of bird blood. Respir. Physiol. 31, 333–343 (1977). 24868 10.1016/0034-5687(77)90076-7

[25] P. Lutz, On the oxygen affinity of bird blood. Am. Zool. 1, 187–198 (1980).

[26] J. F. Storz, Hemoglobin-oxygen affinity in high-altitude vertebrates: Is there evidence for an adaptive trend? J. Exp. Biol. 219, 3190–3203 (2016). 27802149 10.1242/jeb.127134 PMC5091379

[27] J. F. Storz, G. R. Scott, Life ascending: Mechanism and process in physiological adaptation to high-altitude hypoxia. Annu. Rev. Ecol. Evol. Syst. 50, 503–526 (2019). 33033467 10.1146/annurev-ecolsys-110218-025014 PMC7540626

[28] K. B. Tate., Circulatory mechanisms underlying adaptive increases in thermogenic capacity in high-altitude deer mice. J. Exp. Biol. 220, 3616–3620 (2017). 28839010 10.1242/jeb.164491 PMC5665433

[29] C. Lenfant, K. Johansen, J. D. Torrance, Gas transport and oxygen storage capacity in some pinnipeds and the sea otter. Respir. Physiol. 9, 277–286 (1970). 5445188 10.1016/0034-5687(70)90076-9

[30] J. U. Meir, W. K. Milsom, High thermal sensitivity of blood enhances oxygen delivery in the high-flying bar-headed goose. J. Exp. Biol. 216, 2172–2175 (2013). 23470665 10.1242/jeb.085282

[31] A. V. Signore., Adaptive changes in hemoglobin function in high-altitude Tibetan canids were derived via gene conversion and introgression. Mol. Biol. Evol. 36, 2227–2237 (2019). 31362306 10.1093/molbev/msz097 PMC6759075

[32] P. J. Butler, D. R. Jones, Physiology of diving of birds and mammals. Physiol. Rev. 77, 837–899 (1997). 9234967 10.1152/physrev.1997.77.3.837

[33] P. J. Butler, A. J. Woakes, Heart rate and aerobic metabolism in Humboldt penguins, Spheniscus humboldti, during voluntary dives. J. Exp. Biol. 108, 419–428 (1984). 6423763 10.1242/jeb.108.1.419

[34] J. U. Meir, T. K. Stockard, C. L. Williams, K. V. Ponganis, P. J. Ponganis, Heart rate regulation and extreme bradycardia in diving emperor penguins. J. Exp. Biol. 211, 1169–1179 (2008). 18375841 10.1242/jeb.013235

[35] G. Froget., Heart rate and energetics of free-ranging king penguins (Aptenodytes patagonicus). J. Exp. Biol. 207, 3917–3926 (2004). 15472022 10.1242/jeb.01232

[36] P. J. Ponganis, Diving mammals. Compr. Physiol. 1, 447–465 (2011). 23737181 10.1002/cphy.c091003

[37] G. L. Kooyman, J. P. Schroeder, D. G. Greene, V. A. Smith, Gas exchange in penguins during simulated dives to 30 and 68 m. Am. J. Physiol. 225, 1467–1471 (1973). 4760462 10.1152/ajplegacy.1973.225.6.1467

[38] P. J. Ponganis., Returning on empty: Extreme blood O_2_ depletion underlies dive capacity of emperor penguins. J. Exp. Biol. 210, 4279–4285 (2007). 18055617 10.1242/jeb.011221

[39] P. J. Ponganis., O_2_ store management in diving emperor penguins. J. Exp. Biol. 212, 217–224 (2009). 19112140 10.1242/jeb.026096 PMC2720999

[40] A. V. Signore, J. F. Storz, Biochemical pedomorphosis and genetic assimilation in the hypoxia adaptation of Tibetan antelope. Sci. Adv. 6, eabb5447 (2020). 32596473 10.1126/sciadv.abb5447 PMC7299627

[41] H. Pan., High-coverage genomes to elucidate the evolution of penguins. Gigascience 8, giz117 (2019). 31531675 10.1093/gigascience/giz117 PMC6904868

[42] R. C. Edgar, MUSCLE: Multiple sequence alignment with high accuracy and high throughput. Nucleic Acids Res. 32, 1792–1797 (2004). 15034147 10.1093/nar/gkh340 PMC390337

[43] L.-T. Nguyen, H. A. Schmidt, A. von Haeseler, B. Q. Minh, IQ-TREE: A fast and effective stochastic algorithm for estimating maximum-likelihood phylogenies. Mol. Biol. Evol. 32, 268–274 (2015). 25371430 10.1093/molbev/msu300 PMC4271533

[44] S. Kalyaanamoorthy, B. Q. Minh, T. K. F. Wong, A. von Haeseler, L. S. Jermiin, ModelFinder: Fast model selection for accurate phylogenetic estimates. Nat. Methods 14, 587–589 (2017). 28481363 10.1038/nmeth.4285 PMC5453245

[45] D. T. Hoang, O. Chernomor, A. von Haeseler, B. Q. Minh, L. S. Vinh, UFBoot2: Improving the ultrafast bootstrap approximation. Mol. Biol. Evol. 35, 518–522 (2018). 29077904 10.1093/molbev/msx281 PMC5850222

[46] Z. Yang, PAML 4: Phylogenetic analysis by maximum likelihood. Mol. Biol. Evol. 24, 1586–1591 (2007). 17483113 10.1093/molbev/msm088

[47] J. Zhang, R. Nielsen, Z. Yang, Evaluation of an improved branch-site likelihood method for detecting positive selection at the molecular level. Mol. Biol. Evol. 22, 2472–2479 (2005). 16107592 10.1093/molbev/msi237

[48] Z. Yang, S. Kumar, M. Nei, A new method of inference of ancestral nucleotide and amino acid sequences. Genetics 141, 1641–1650 (1995). 8601501 10.1093/genetics/141.4.1641 PMC1206894

[49] J. P. Bielawski, Z. Yang, A maximum likelihood method for detecting functional divergence at individual codon sites, with application to gene family evolution. J. Mol. Evol. 59, 121–132 (2004). 15383915 10.1007/s00239-004-2597-8

[50] C. J. Weadick, B. S. W. Chang, An improved likelihood ratio test for detecting site-specific functional divergence among clades of protein-coding genes. Mol. Biol. Evol. 29, 1297–1300 (2012). 22319160 10.1093/molbev/msr311

[51] A. Waterhouse., SWISS-MODEL: Homology modelling of protein structures and complexes. Nucleic Acids Res. 46, W296–W303 (2018). 29788355 10.1093/nar/gky427 PMC6030848

[52] P. Benkert, M. Biasini, T. Schwede, Toward the estimation of the absolute quality of individual protein structure models. Bioinformatics 27, 343–350 (2011). 21134891 10.1093/bioinformatics/btq662 PMC3031035

[53] E. Krissinel, K. Henrick, Inference of macromolecular assemblies from crystalline state. J. Mol. Biol. 372, 774–797 (2007). 17681537 10.1016/j.jmb.2007.05.022

[54] C. Natarajan., Expression and purification of recombinant hemoglobin in Escherichia coli. PLoS One 6, e20176 (2011). 21625463 10.1371/journal.pone.0020176 PMC3098879

[55] C. Natarajan., Epistasis among adaptive mutations in deer mouse hemoglobin. Science 340, 1324–1327 (2013). 23766324 10.1126/science.1236862 PMC4409680

[56] S. Claramunt, J. Cracraft, A new time tree reveals Earth history’s imprint on the evolution of modern birds. Sci. Adv. 1, e1501005 (2015). 26824065 10.1126/sciadv.1501005 PMC4730849

