# Supplementary material for: Evolved increases in hemoglobin-oxygen affinity and the Bohr effect coincided with the aquatic specialization of penguins
Source: Proc Natl Acad Sci U S A. 2021 Mar 22;118(13):e2023936118. doi: 10.1073/pnas.2023936118 (PMC8020755; doi:10.1073/pnas.2023936118)
Supplement: Supplementary File [file pnas.2023936118.sapp.pdf]

PNAS
www.pnas.org

Supplementary Information for
Evolved increases in hemoglobin-oxygen affinity and Bohr effect coincided with the aquatic specialization of penguins.

Anthony V. Signore[a], Michael S. Tift[b], Federico G. Hoffmann[c,e], Todd. L. Schmitt[d], Hideaki Moriyama[a], Jay F. Storz[a*]

[a]School of Biological Sciences, University of Nebraska, Lincoln, NE 68588, USA.

[b]Department of Biology and Marine Biology, University of North Carolina, Wilmington, NC 28403, USA

[c]Department of Biochemistry, Molecular Biology, Entomology, and Plant Pathology, Mississippi State University, MS 39762, USA

[d]Veterinary Services, SeaWorld of California, San Diego, CA 92109, USA

[e]Institute for Genomics, Biocomputing and Biotechnology, Mississippi State University, Starkville, MS 39762

*Correspondence to: Anthony V. Signore
Email: anthony.signore@unl.edu

This PDF file includes:

Figures S1 to S4
Tables S1 to S3
SI References

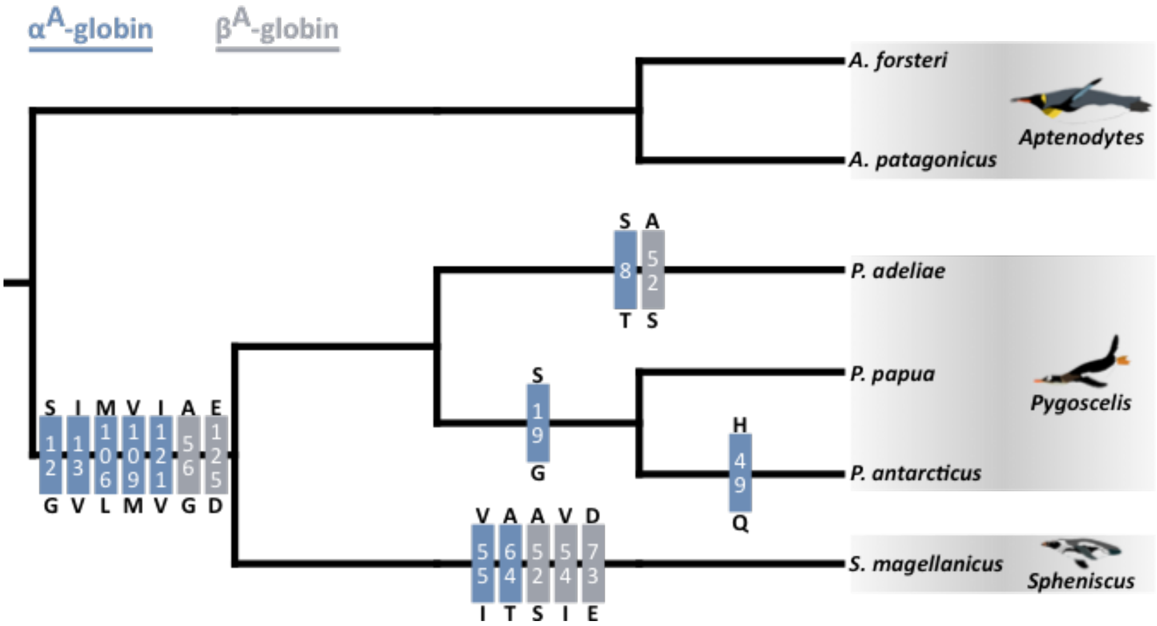

Fig. S1. Amino acid substitutions in the α- and β-globin chains of the six penguin species that were used in the experimental analysis of Hb function. Blue and grey numbered boxes represent amino acid positions in the α-globin and β-globin chains, respectively. Amino acids listed above and below each position represent the ancestral and derived amino acid state, respectively. The penguin phylogeny is adapted from Pan et al. (1).

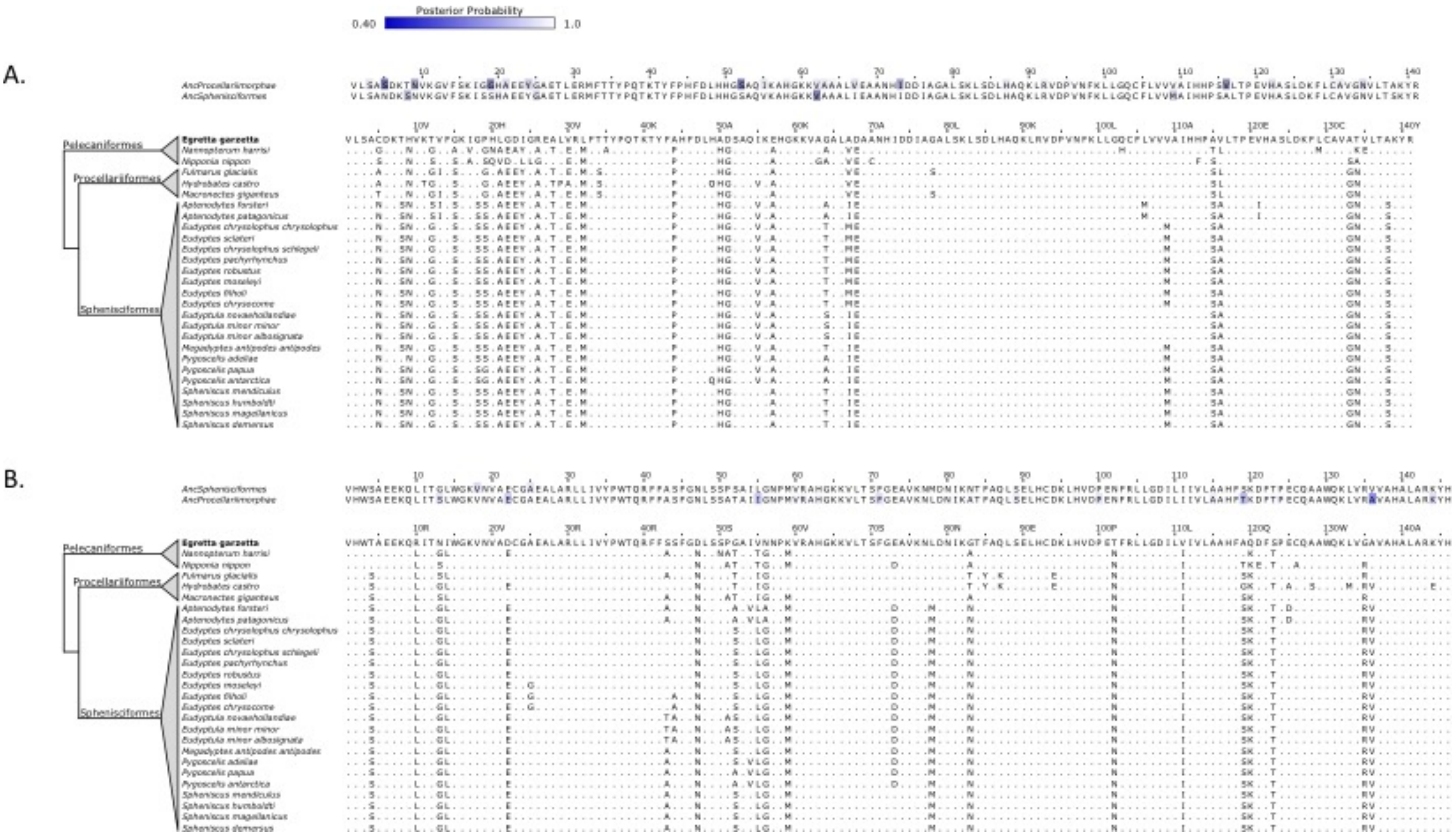

Fig. S2. Amino acid sequences for waterbird (A) α-globin and (B) β-globin chains. Estimated ancestral sequences for AncProcellariimorphae and AncSphenisciformes are shown above the native protein sequences. Shading on the ancestral protein sequences represent the posterior probability of each ancestral character estimate, where darker coloration represents lower posterior probability (see Figures S3 and S4 for more detail). Dots represent identity with *Egretta garzetta*.

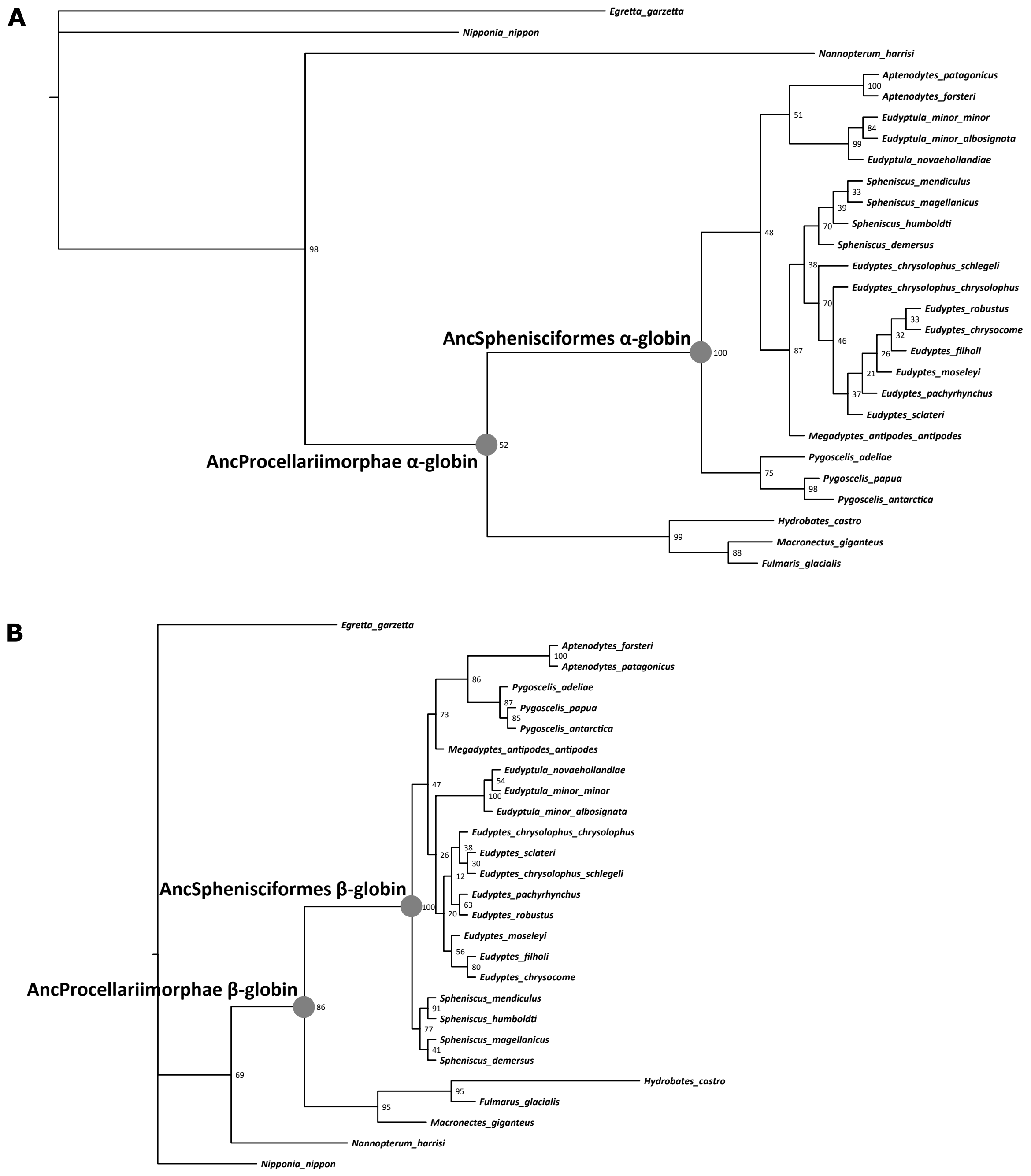

Fig. S3. Estimated maximum likelihood phylogenies of waterbird (A) α-globin and (B) β-globin genes. Filled circles represent nodes for which ancestral sequences were reconstructed. Node labels represent percentage bootstrap support.

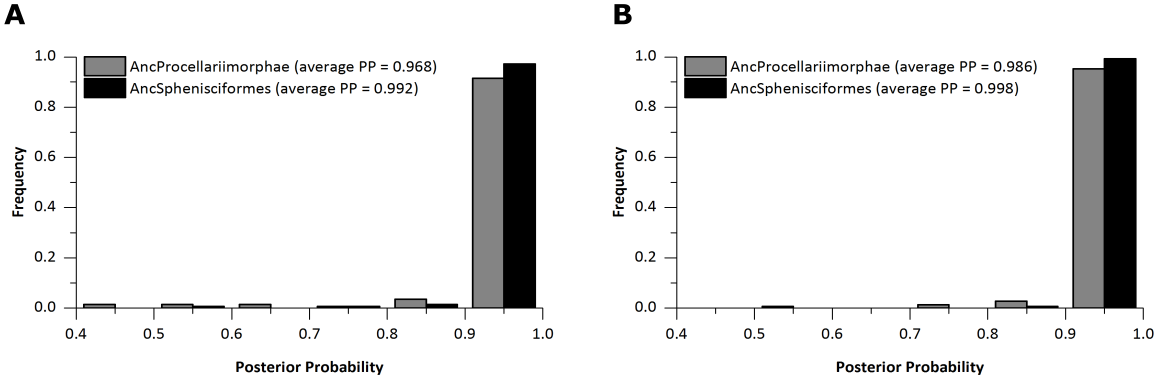

Fig. S4. Distributions of site-specific posterior probabilities for the (A) α-globin and (B) β-globin genes of the estimated penguin ancestor (AncSphenisciformes) and the last common ancestor penguins shared with Procellariiformes (AncProcellariimorphae).

Table S1. Oxygen affinities (P_{50}, torr) of penguin whole-blood and native Hbs with their sensitivity to allosteric effectors at 37°C in 0.1 M HEPES buffer at pH 7.4.

Species	[a]Whole-Blood P_{50}	[b]Whole-blood n_{50}	[a]Stripped P_{50}	[a]Stripped $\log P_{50}$	[b]Stripped n_{50}	[c]Cofactor Effect	[d]Bohr Coefficient ($\Delta \log P_{50}/\Delta pH$)	
							Stripped	KCl + IHP
A. forsteri	31.77±1.19	2.67±0.03	3.25±0.16	0.51±0.02	1.48±0.13	0.89	-0.282±0.054	-0.502±0.009
A. patagonicus	38.10±0.99	2.79±0.14	4.46±0.12	0.65±0.01	1.24±0.03	0.83	-0.120±0.036	-0.554±0.133
P. adeliae	33.16±3.73	2.37±0.11	3.50±0.36	0.54±0.04	1.44±0.12	0.82	-0.300±0.124	-0.625±0.037
P. papua	31.74±0.51	2.72±0.13	3.54±0.60	0.55±0.07	1.59±0.14	0.83	-0.178±0.221	-0.661±0.055
P. antarcticus	30.37±1.60	2.45±0.35	3.80±0.08	0.58±0.01	1.42±0.05	0.66	-0.190±0.035	-0.454±0.105
S. magellanicus	34.97±1.30	3.27±0.38	3.36±0.07	0.53±0.01	1.64±0.03	0.84	-0.190±0.022	-0.365±0.035

[a]P_{O2} at half-saturation (torr)
[b]Hill's cooperativity coefficient
[c]$\Delta \log P_{50}$([KCl+IHP] – Stripped)
[d]Purified Hb, pH range 7.2 to 7.6

Table S2. Branch-site model statistics for reconstructed ancestral Sphenisciformes α- and β-globin genes. NS = Not significant.

Model	np	-lnL	Estimates	LRT
α-globin Model A null	55	-1454.78	Background: ω_0=0.04, ω_1=1.00, ω_{2a}=0.04, ω_{2b}=1.00<br>Foreground: ω_0=0.04, ω_1=1.00, ω_{2a}=1.00, ω_{2b}=1.00<br>p_0=0.63, p_1=0.29, p_{2a}=0.08, p_{2b}=0.03	
α-globin Model A alt (ω1 > 1)	56	-1454.76	Background: ω_0=0.05, ω_1=1.00, ω_{2a}=0.04, ω_{2b}=1.00<br>Foreground: ω_0=0.05, ω_1=1.00, ω_{2a}=2.50, ω_{2b}=2.50<br>p_0=0.68, p_1=0.26, p_{2a}=0.04, p_{2b}=0.02	NS
β-globin Model A null	55	-1359.51	Background: ω_0=0.03, ω_1=1.00, ω_{2a}=0.03, ω_{2b}=1.00<br>Foreground: ω_0=0.03, ω_1=1.00, ω_{2a}=1.00, ω_{2b}=1.00<br>p_0=0.85, p_1=0.15, p_{2a}=0.00, p_{2b}=0.00	
β-globin Model A alt (ω1 > 1)	56	-1359.51	Background: ω_0=0.03, ω_1=1.00, ω_{2a}=0.03, ω_{2b}=1.00<br>Foreground: ω_0=0.03, ω_1=1.00, ω_{2a}=1.00, ω_{2b}=1.00<br>p_0=0.85, p_1=0.15, p_{2a}=0.00, p_{2b}=0.00	NS

Table S3. Clade model statistics for reconstructed ancestral Sphenisciformes α- and β-globin genes. NS = Not significant.

Model	np	-lnL	Site Class	Proportion	ω	ω Branch Type 0	ω Branch Type 1	LRT
			0	0.53	0.00			
α-globin Model 2a_rel	56	-1452.71	1	0.11	1.00			
			2	0.36	0.36			
			0	0.69		0.04	0.04	
α-globin Model C	57	-1451.82	1	0.15		1.00	1.00	NS
			2	0.17		1.01	0.00	
			0	0.65	0.03			
β-globin Model 2a_rel	56	-1359.51	1	0.15	1.00			
			2	0.2	0.03			
			0	0.43		0.03	0.03	
β-globin Model C	57	-1358.49	1	0.15		1.00	1.00	NS
			2	0.42		0.06	0.00	

SI References

1. H. Pan, T.L. Cole, X. Bi, M. Fang, C. Zhou, Z. Yang, D.T. Ksepka, T. Hart, J.L. Bouzat, L.S. Argilla, M.F. Bertelsen, High-coverage genomes to elucidate the evolution of penguins. *GigaScience*, 8(9), p.giz117 (2019).
